# An evaluation of the Hear Glue Ear mobile application for children aged 2–8 years old with otitis media with effusion

**DOI:** 10.1177/2055207620966163

**Published:** 2020-10-25

**Authors:** Surina Fordington, Tamsin Holland Brown

**Affiliations:** 1 University of Cambridge School of Clinical Medicine, Addenbrooke's Hospital, Cambridge, UK; 2 Cambridge Community Services, Brookfields Hospital, Cambridge, UK

**Keywords:** Otitis media with effusion, OME, mobile application, speech, language development, audiology, child, hearing

## Abstract

**Objectives:**

To evaluate the acceptability and usability of the Hear Glue Ear mobile application to guide families and support speech and language development in children with otitis media with effusion (OME). To assess the validity of the app’s game-based hearing test to estimate changes in hearing levels between audiology appointments.

**Method:**

This evaluation examined 60 children aged 2–8 with and without OME, attending Cambridge Community Audiology clinics. Children’s performance in the app’s hearing test was compared to their pure tone average (PTA) obtained in clinic. Children and caregivers completed questionnaires after their first interaction with the app, and after one week of using it at home. 18 clinicians completed anonymous questionnaires after trialling the app.

**Results:**

Results from the app’s hearing test show a significant correlation with clinic PTA values ($r\left(22\right)=-0.656,p=0.000251$). 73.1% of caregivers supported their child using the app regularly and 85% thought it enabled them to give more accurate reports to clinicians. After one week, 87.0% of families downloaded and used the app at home, and 85.7% of these felt it provided strategies to help their child. 100% of children liked the app and 93.3% found it easy to use. 77.8% of clinicians supported patients using the app regularly.

**Conclusions:**

Hear Glue Ear is acceptable to children, caregivers and clinicians as part of OME management. The app’s hearing test provides a valid estimate of fluctuating hearing levels. Hear Glue Ear is a free, accessible and family-centred intervention to provide trusted information and support development, as NICE guidance recommends.

## Introduction

### Otitis media with effusion: Background and aetiology

Otitis media with effusion (OME), also known as glue ear, is the leading cause of childhood hearing loss worldwide and affects an estimated 1 in 5 pre-school children in the UK at any one time.^1^ OME is caused by accumulation of serous fluid in the middle ear, often secondary to ear or upper respiratory tract infections. This can impair the transfer of sound to the ossicles, resulting in a conductive hearing loss. Children with cleft palate, skeletal dysplasia and other conditions such as Down’s Syndrome are at increased risk of developing OME.^2^ In most children, OME resolves spontaneously after an average of 6–10 weeks,^3^ but in some cases it may result in a chronic hearing loss which can significantly impact quality of life.^4^

**Figure 1.**
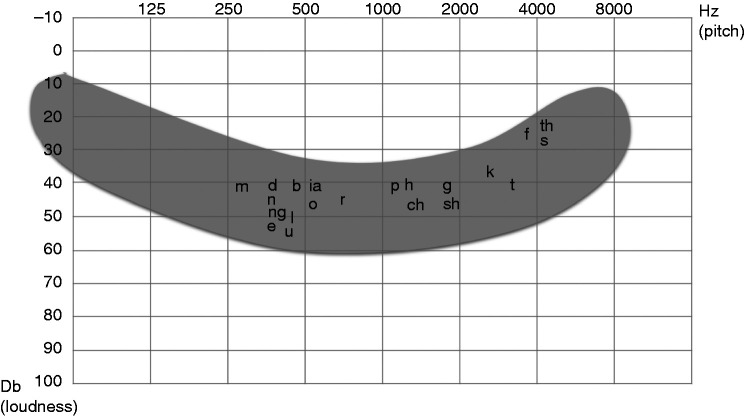
Speech ‘banana’ showing the range of frequencies used in everyday speech. The letters correspond to individual sounds used in speech. In OME-associated hearing loss, lower frequency sounds are commonly compromised.

Chronic OME is associated with long term speech and language impairments, since knowledge about syntactic structure, which is critical for language learning, may be impaired by reduced exposure to acoustic-phonetic stimuli.^5^ In particular lower frequency inputs that correlate to many speech sounds are often compromised, as illustrated in Figure 1.^6^ In addition, the ages with highest prevalence of OME coincide with a critical window for speech and language development.^7^ As a result, auditory processing and learning can be adversely affected, and are often associated with social and behavioural difficulties which can persist into teenage years.^8–11^ The diagnosis of OME is frequently overlooked due to its non-specific presentation, resulting in many children being prematurely labelled with behavioural or educational issues. The National Institute for Health and Care Excellence (NICE) informs that hearing loss from OME can create social challenges for children and place strain on families.^12^ This is corroborated by feedback during PPV (Patient and Public Voice) research preceding this study, which revealed anecdotally that many parents struggle to manage their child’s behaviour while their hearing is reduced from OME. Often this is due to challenges differentiating between whether their child needs clear parental boundaries or is simply exhausted from struggling to listen and communicate at school.

### Management of OME

**Figure 2.**
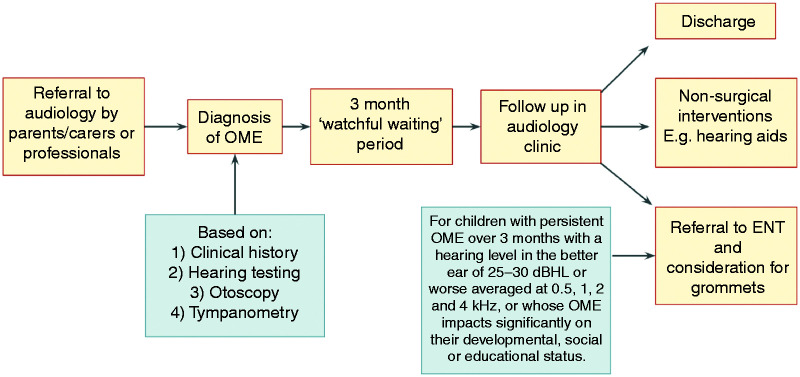
Current management of OME in the UK.^12^

As summarised in Figure 2, the UK standard for OME management is an initial assessment by a paediatric audiologist, followed by a 3 month ‘watchful waiting' period before a repeated hearing assessment.^12^ If OME persists after this interval, the child may be referred to Ear, Nose and Throat (ENT) for consideration of hearing aids or ventilation tubes (grommets). The period of ‘watchful waiting’ for spontaneous remission combined with referral delays can result in children with persistent OME spending a significant period of time with reduced hearing. In addition, OME has a high recurrence rate, which results in many children being discharged from follow-up services during a temporary period of resolution, only to deteriorate again. Review of the current literature highlights a need for additional support between appointments, in order to minimise delays in speech and language development. 60% of parents of children with mild or moderate hearing loss report needing more support for their child.^13^ Actively supporting speech and language development following OME diagnoses could improve learning and behaviour in the long term. Behind-the-Ear (BTE) hearing aids during this period are unpopular due the fluctuating nature of the hearing loss and the challenge of selectively amplifying low frequency sounds.^14^,^15^ As an alternative or adjunct, NICE guidance highlights the need for “educational and behavioural strategies to minimise the impact of hearing loss” and tools to guide families on how to support their child from home.^12^ In particular, during the current COVID-19 pandemic, face-to-face appointments and surgical interventions are restricted, so there is demand from families and clinicians to facilitate continuity of care by supporting children’s development and auditory processing remotely. The Hear Glue Ear app addresses this need by providing focused activities to support children as well as trusted information to guide families while their child’s hearing is reduced from OME.

### The use of mobile health apps

Health applications are an increasingly popular and cost-effective way for patients to monitor, report and self-manage health conditions from home.^16^ The role of mobile applications for healthcare (mobile health or mHealth) is rapidly expanding, with regulation and review offered by the NHS App Library and ORCHA (Organisation for the Review of Care and Health Applications), in order to offer doctors guidance and security with recommending apps. A number of applications exist for adult audiology (e.g. hearWHO by the World Health Organisation, uHear by Unitron), but there are currently very few high quality apps addressing childhood hearing loss.^17^ This is surprising considering the increasing presence of technology in schools and evidence showing that children’s engagement with speech and language support is increased when it is in electronic form.^18^ Barriers to the use of mobile applications for childhood hearing loss may include accessibility and compliance in children and parental concerns about screen time.^19^ There are concerns that increased screen time may adversely impact speech development in young children,^20^ although this association has been contradicted by a recent study.^21^ A key aim of this current study is to assess whether these concerns pose significant barriers to the uptake of Hear Glue Ear, which is designed to support speech and language development as well as auditory processing and listening skills.

### The Hear Glue Ear application

**Figure 3.**
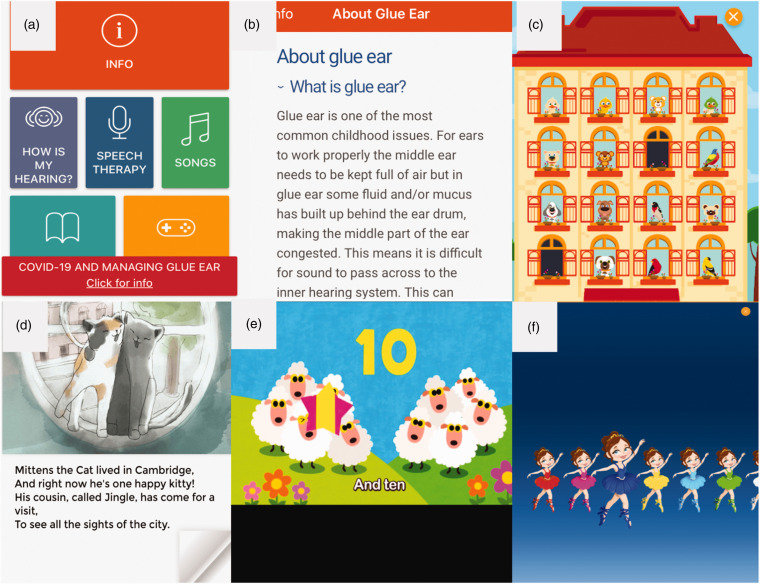
Sections of the ‘Hear Glue Ear’ app: (a) Home panel; (b) Information; (c) ‘How’s My Hearing?’ hearing test; (d) Audiobook; (e) Counting song; (f) ‘Getting Dressed’ listening game.

Hear Glue Ear was designed using a multidisciplinary and user-centred design (UCD) approach following recommendations from speech and language therapists, audiologists, ENT surgeons, paediatricians, parents and teachers of the deaf. The user interface is designed to be informative and accessible for children and caregivers and is displayed in Figure 3. The app is designed for children between the ages of 2 and 8 years, reflecting the ages with highest OME prevalence.^3^ The features of the app aim to meet NICE recommendations for supporting speech and language development during management of OME^3^ and to address specific user needs, as summarised in Table 1. It has received an ORCHA quality mark badge and has recently been highly commended by NICE as a shared learning example.^22^ Hear Glue Ear is free to download from Apple and Android app stores, thus addressing a demand for affordable support for children with hearing loss. This was prioritised by the makers of the app since studies across several countries have shown that the impact of OME is greater in lower income families.^23^,^24^

**Table 1.** Summary of the user requirements addressed by the Hear Glue Ear application.

**User requirement**	**App feature**	**Specific features which meet requirements**
Informing parents about OME and its management	Information section	Information and frequently asked questions (FAQs) about OME and its management, provided by National Deaf Children’s Society (NDCS). Information is concise and easy for parents to access remotely and reduces the need for environmentally damaging paper leaflets currently used.
Monitoring a child’s hearing from home	‘How’s My Hearing?’ hearing test	Game-based hearing test. Uses 500–4000 Hz frequency and 20–70 dB volume with warble and pure tone audiometry. Correctly and incorrectly heard sounds are displayed on a matrix-style graph for parents and clinicians to view.
Identifying trends in OME-associated hearing loss	Analytics section	Results in the ‘How’s My Hearing?’ hearing test are displayed over time in a line graph to provide an estimate of trends in hearing loss, which is known to fluctuate in OME.
Developing auditory processing skills	Audiobooks	Audiobooks enable children to match auditory cues with visual representations throughout. Questions at the end of each story aim to develop skills around auditory processing and auditory memory.
Exposure to acoustic-phonetic stimuli	Songs	The songs, provided by Pinkfong educational videos, focus on phonetics and rhyming to improve children’s exposure to the full enrichment of speech sounds.
Following auditory instructions	‘Getting Dressed’ game	The game involves children listening to and following auditory instructions and aims to develop listening and auditory processing skills and to practise building on auditory cues in a relaxed home environment.
Access to tailored speech support	Speech and Language Therapy section	The portal enables speech and language therapists to upload personal speech support videos for children. The therapist can view when the child has seen the video and upload the next one.
Encouraging parental engagement with their child’s speech and language development	All of the above	Clinician recommendation of the app and clear guidance in the app encourage active and supported involvement of parents with their child’s hearing loss management, which has been shown to improve long term speech and language outcomes.^25^

### The ‘How’s My Hearing?’ game-based hearing test

The Hear Glue Ear application contains a game-based hearing test, designed to provide parents with an estimate of their child’s hearing level between audiology appointments. It is known that hearing loss due to OME fluctuates, and as such it is often difficult for parents and teachers to identify when a child’s challenging behaviour is due to frustration and fatigue from struggling to hear. This challenge may be so significant that some children with OME can be initially referred for assessment of autism and learning disabilities. The Hear Glue Ear app’s hearing test offers parents an estimate of their child’s hearing level between appointments, in order to gain confidence with managing their behaviour. It is not intended to be diagnostic of hearing loss or to replace clinical audiology assessment.

**Figure 4.**
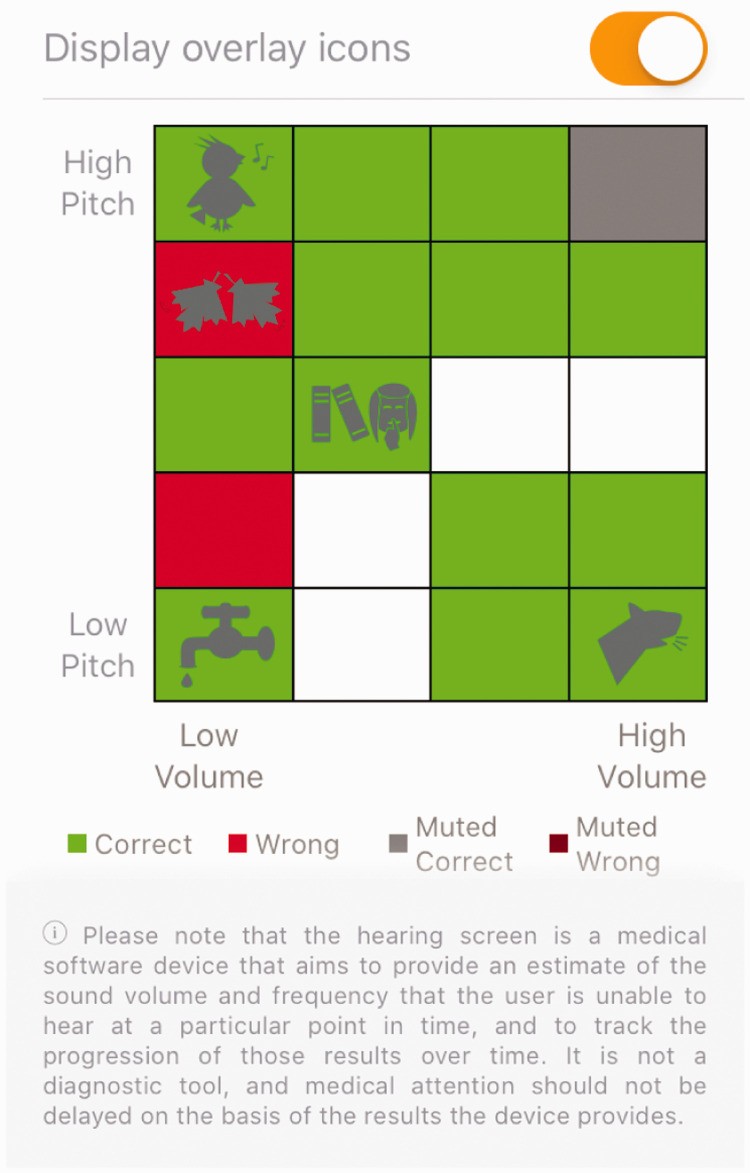
User interface showing the results of the hearing screen, which can be accessed by parents and clinicians. The green bars represent sounds correctly identified, the red bars represent sounds not identified and the grey bar represents muted controls correctly identified. The white boxes indicate that no sound was presented. The parent may choose to display overlay icons which attempt to correlate their child’s performance in the listening game with everyday sounds they may be struggling to hear.

‘Gamification’ is the process of applying game-design elements into a non-game context in order to engage users and promote positive behavioural change.^26^ In paediatric patients, gamification within mHealth has been shown to increase engagement and compliance with healthcare-related activities.^27^,^28^ The game-based hearing test within the Hear Glue Ear app presents children with a series of pure or warble tones at frequencies and volumes comparable to those used in standard pure tone audiometry tests (frequencies of 500 Hz, 1 kHz, 2 kHz and 4 kHz and volumes in the range 20–80 dB SPL). These are presented within a game design, where the child is shown cartoon animals in windows and must tap on an animal and wait for a sound. The test uses a yes/no paradigm where the child selects an option depending on whether or not they heard the sound, which is recorded by the app. There is no positive or negative reinforcement following a child’s response to avoid biasing future responses. The frequency and volume of each sound is randomly distributed and varied between tests in order to avoid sequence learning, and there are always two mute animals per game to discourage users from arbitrarily selecting ‘yes’ responses. The number of correct and incorrect answers per attempt is visible to parents and clinicians (with parental consent) in the analytics section of the app as matrix of volume against frequency, as shown in Figure 4, and may also be compared to previous attempts.

**Figure 5.**
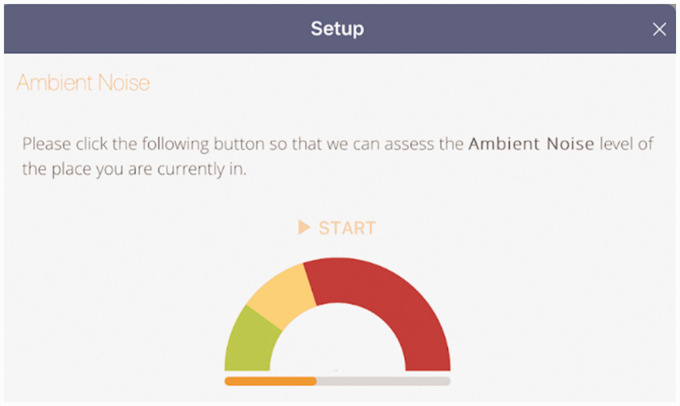
User interface showing the assessment of the ambient noise level, which must be below 35 dB for the test to commence.

**Figure 6.**
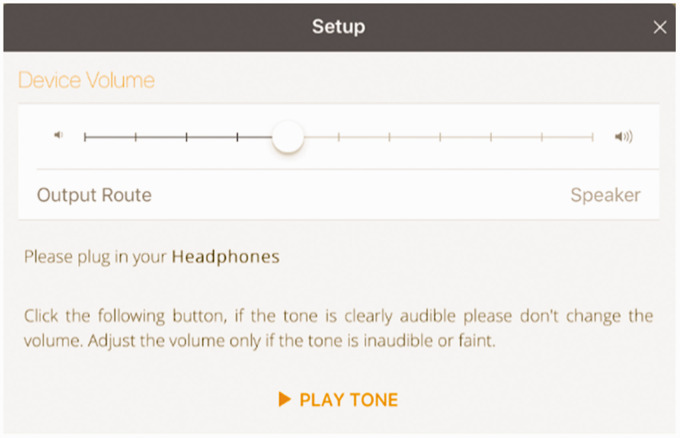
User interface showing establishment of the hearing screen volume level, which is relative to the minimum audible level of an adult with normal hearing.

During the app set-up, the test environment is calibrated and the hearing test is allowed to proceed if the ambient noise is 35 dB or less, as shown in Figure 5. In order to control for variations in volume level between device speakers, tone volumes are standardised by adjusting to the minimum sound audible to the operating adult, assuming that they have normal hearing, as shown in Figure 6. Provided the same adult supervises the child with each use, ideally in the same environment or same room at home, the results may be compared to estimate trends in hearing over time.

### OME and Down’s Syndrome

Numerous studies have shown that children with Down’s Syndrome have a higher prevalence of OME.^29^ This is thought to result from differences in the anatomy of the Eustachian tube and increased susceptibility to middle ear infections. Children with Down’s Syndrome frequently experience challenges with learning, speech and language, and prolonged hearing impairment due to chronic OME can exacerbate these developmental delays.^30^ Furthermore, there are significant discrepancies in access to hearing care across the UK which can create variation in the speed of identification and the level of speech and language support received.^31^ The Hear Glue Ear app could potentially provide remote speech, language and listening support regardless of location for children with Down’s Syndrome, in order to minimise developmental delay.

## Methods and materials

Ethical approval was obtained from Wales REC 7 (IRAS ID: 262154).

### Participants

The study involved 60 children (male n = 31, female n = 29) aged 2–8 years old and their accompanying caregiver(s), attending Cambridge Community Paediatric Audiology clinics. All children were attending the clinic with suspected or previously diagnosed OME. Following assessment, 25 children had an OME diagnosis and 35 did not. Additionally, 6 children had a diagnosis of Down’s Syndrome and no current OME diagnosis. 1 child had an unspecified learning disability other than Down’s Syndrome. The study also included 18 clinicians working in Cambridgeshire Community Services (CCS), which consisted of paediatricians (n = 11), audiologists (n = 5), a GP and a paediatric psychologist.

### Experimental design

**Figure 7.**
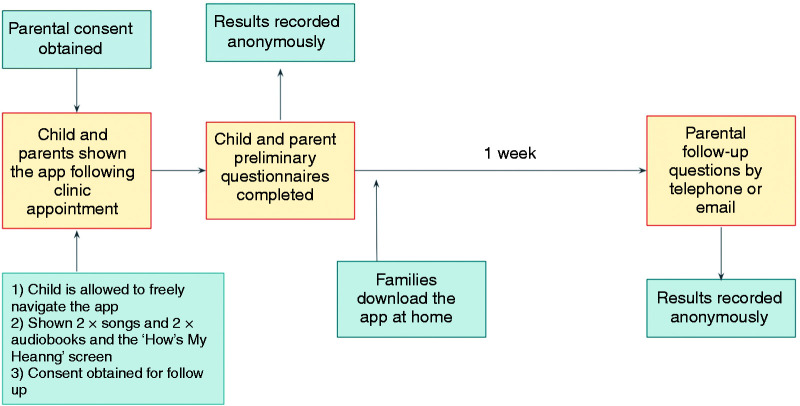
Summary of the experimental protocol for the study.

The experimental protocol is summarised in Figure 7. Families who consented were enrolled in the study following their audiology clinic appointment. The written parental consent form is included in online Appendix A. Families were offered to return on a different day if they wanted time to consider involvement in the research study; however all of the participating families chose to take part in the study on the same day following their clinic appointment. Children and their caregivers were shown the Hear Glue Ear app by a researcher in a standard clinic room on a 9.7” Apple iPad. Ambient noise was restricted to within the sound level meter limits specified on the app and the tone volume was set according to the hearing level of the researcher (who remained constant throughout the study).

Children were allowed to navigate the app by themselves from the home screen and were shown 2 audiobooks and 2 songs. They were also shown the ‘How’s My Hearing?’ hearing test and encouraged to complete it once. Children were presented with an oral questionnaire on their impressions of the app, whilst their accompanying caregiver was given a written questionnaire to complete. One week later, families who had consented were followed up via telephone or email in order to assess level of uptake of the app and families’ experience of using the app at home. Not every family completed all of the stages within the protocol due to limited time availability, children’s concentration and compliance and families’ responsiveness to follow-up. Clinicians were shown the app in person by a researcher or via an emailed link and were sent a questionnaire which they completed and returned anonymously, in order to minimise bias.

### Comparison of the app’s hearing screen with audiometry results

In order to assess the validity of the app’s game-based hearing test, the results obtained on the app were compared with the pure tone audiometry results obtained by each child in clinic. The child was encouraged to complete the app’s hearing test on their first interaction with the app and the percentage score of correct answers was recorded by the researcher. The app’s hearing test presents sounds of 500, 1000, 2000 and 4000 Hz frequencies and between 20 and 70 dB pitch. Each child’s hearing level in the audiology clinic was assessed using pure tone audiometry. This presents each child with sounds at 500, 1000, 2000 and 4000 Hz and the minimum audible volume in decibels is recorded. The pure tone average (PTA) is the mean of the minimum audible volumes in decibels heard at all 4 frequency levels. Thus, the lower the PTA value, the ‘better’ a child’s hearing level. The PTA obtained in audiology clinic was compared to the percentage score obtained in the app’s hearing screen. Since all families chose to take part in the study on the same day as their appointment, there was a maximum delay of 30 minutes between audiometry assessment in clinic and completing the app’s hearing test. Transient fluctuations in hearing level over such a short period are unlikely to affect results and therefore need not be taken into consideration in statistical analysis.

### Preliminary questionnaire

The children’s questionnaires consisted of 15 yes/no questions assessing the acceptability and accessibility of the app. The caregiver questionnaire consisted of 18 written questions using a Likert scale (response agreement was graded from 1–5) to assess accessibility, acceptability and usability for families. Free text spaces were available to express additional views on app improvement. The first 13 questions of the caregiver and clinician questionnaires follow the standard SUS (System Usability Scale) for evaluation of an application, as used in previous app evaluations.^32^ The child and caregiver preliminary questionnaires are included in online Appendix B.

### Follow-up questionnaire

The follow-up questionnaire consisted of 12 mixed yes/no and free-text questions. These assessed levels of uptake, engagement, use of specific features and feedback on the appropriateness of the app for the home environment. The carer follow up questionnaires are included in online Appendix B.

### Clinicians’ questionnaire

The anonymous questionnaires given to clinicians consisted of 19 questions using a Likert scale (1–5 grading) and free text space to assess the acceptability to healthcare professionals of including the app within clinical pathways. The clinician questionnaire is included in online Appendix B.

### Mobile Application Rating Scale (MARS)

10 adults (consisting of clinicians working in CCS and caregivers whose children were attending paediatric audiology services) were shown the app and asked to complete a MARS questionnaire, which is included in online Appendix C. This is a verified app-specific evaluation tool consisting of 23 questions using a Likert scale (graded 1–5).^33^ The ‘app mean quality score’ is calculated as a mean average of the responses to 19 questions in the categories ‘engagement’, ‘functionality’, ‘aesthetics’, and ‘information quality’, and an ‘app subjective quality score’ is calculated as a mean average of the responses to 4 questions assessing subjective opinions of the app.

### Statistical analysis

A Spearman rank correlation coefficient was used to calculate the relationship between percentage scores obtained in the app’s hearing test and the pure tone average obtained in the audiology clinic for each child (where p < 0.05 is significant). The results of each questionnaire were recorded as percentages of overall responses, with no further statistical analysis performed.

## Results

### Participants

60 children between the ages of 2 and 8 years old participated in the study. Near equal gender distribution (51.7% (31/60) male; 48.3% (29/60) female) was ensured to avoid skew, since mobile device use has been shown to vary with gender.^34^ The modal age of participants was 4 years old, which reflects the age of highest prevalence of OME.^7^ The inclusion of children with both glue ear (41.7% (n = 25)) and normal hearing (58.3% (n = 35)) diagnoses in the study group reflected the fluctuating nature of hearing loss in glue ear, and enabled assessment of the app’s usability during times of both impaired and normal hearing, both of which are seen in the watchful waiting period.

### Validity of the ‘How’s My Hearing’ screen

**Figure 8.**
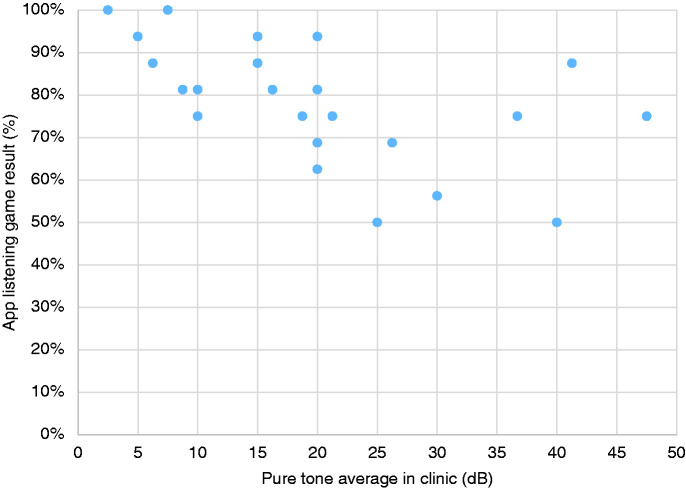
Comparison of percentage score obtained in the app’s hearing screen with the pure tone average (PTA) obtained in clinic for each child ($r\left(22\right)=-0.656,\;p=0.000251$).

There was a significant negative correlation between the percentage score obtained in the app’s game-based hearing test and the pure tone average (PTA) obtained in clinic for each child ($r\left(22\right)=-0.656,p=0.000251$). The higher the percentage score in the app, the better the theoretical hearing level. The lower the PTA, the better their hearing level. This negative correlation shows that on the whole children who performed better in the audiology test in clinic also performed better in the app’s hearing screen. This is shown in Figure 8.

### Children’s preliminary questionnaire

The responses of children when orally presented with a questionnaire following their first use of the app are summarised in Table 2.

**Table 2.** Child responses to the oral questionnaire given immediately after being shown the app. % shows the percentage of responses where a child clearly responded ‘yes’ and excludes ‘no’ or neutral responses.

Response	% (number who agreed/total)
Acceptability	
Liked using the app	100 (18/18)
Found the app fun	100 (18/18)
Accessibility	
Thought the app was easy to use	93.3 (14/15)
Could use the app by themselves without the help of an adult	73.3 (11/15)
Thought their friends would be able to use the app	100 (13/13)
Thought other children would like the app	100 (13/13)

### Caregiver preliminary questionnaire

**Figure 9.**
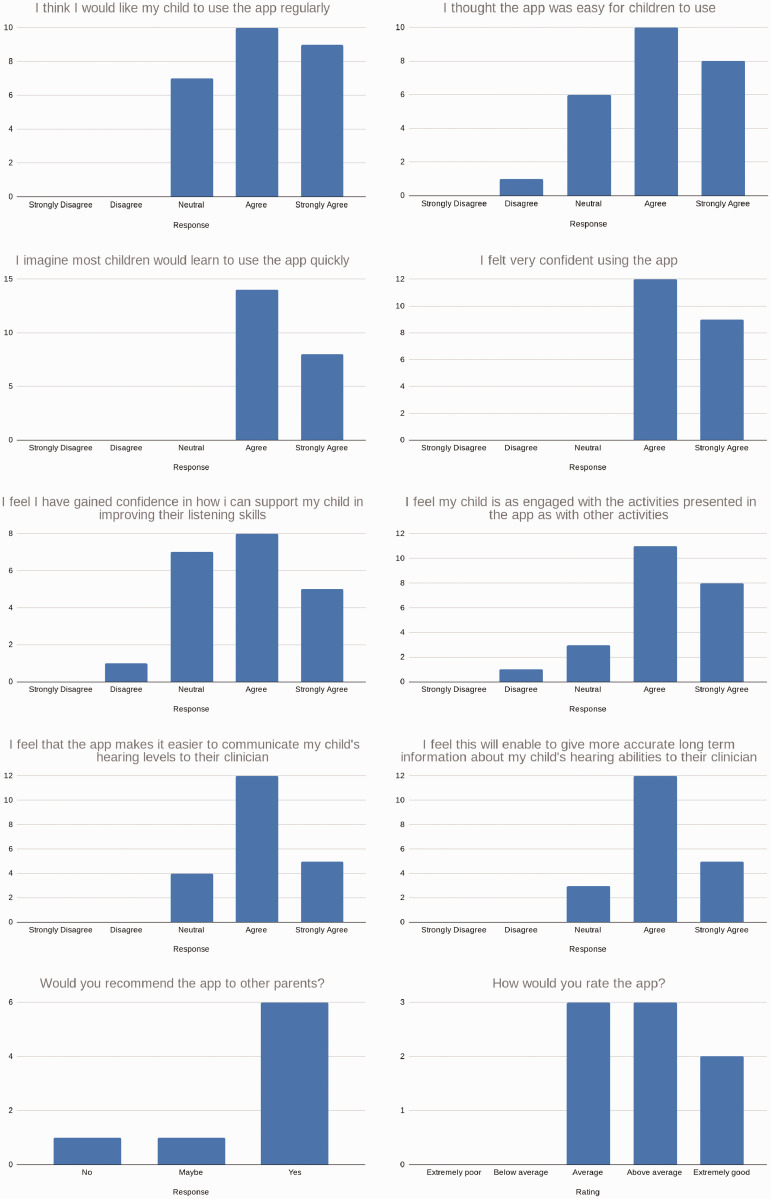
Panel of responses to the caregiver questionnaire.

The responses of caregivers to the preliminary questionnaire are summarised in Table 3 and the agreement distributions for each question are shown graphically in Figure 9.

**Table 3.** Caregiver responses to the written questionnaire given immediately after being shown the app. % shows the percentage of responses where ‘agree’ or ‘strongly agree’ were selected (total number of responses minus ‘neutral’, ‘disagree’ or ‘strongly disagree’).

Response	% (number who agreed/total)
Acceptability	
Would like their child to use the app regularly	73.1% (17/26)
Would recommend the app to other families	87.5% (7/8)
Accessibility	
Thought most children and adults would learn to use the app quickly	100% (21/21)
Felt confident using the app themselves	100% (21/21)
Thought the app was easy for children to use	72% (18/25)
Thought their child could use the app without assistance	59.1% (13/22)
Thought that they themselves could use the app without the support of a technical person	95.8% (23/24)
Felt that they did not need to learn anything new before using the app	85% (17/20)
Usefulness	
Felt they had gained confidence in how to support their child improve their listening skills	61.9% (13/21)
Felt that the app made it easier to report their child’s hearing levels to the clinician	81% (17/21)
Felt it would enable them to give more accurate long term information about their child's hearing to clinicians	85% (17/20)
Felt their child was as engaged with the activities present in the app as with other activities	82.6% (19/23)

### Caregiver follow-up questionnaires

The responses of carers to the one-week follow- up questionnaire are summarised in Table 4. The most frequently used feature of the app was the screening test (used by 93.3% (14/15) families who responded).

**Table 4.** Caregiver responses to the follow-up questionnaire given after using the app for one week at home. % shows the percentage of responses where ‘yes’ was clearly stated, and excludes ‘no’ or neutral responses.

Response	% (number who agreed/total)
Uptake	
Had downloaded the app	87.0% (20/23)
Had used the app between appointments	73.9% (17/23)
Usefulness	
Understood the purpose of the app	100% (16/16)
Felt the app provided strategies to help their child	85.7% (12/14)
Thought that the app helped to support their child whilst their hearing was reduced from glue ear	61.5% (8/13)
Accessibility	
Found the app easy to use	100% (8/8)
Thought their child found the app easy to use	100% (8/8)
Acceptability	
Felt their child enjoyed using the app	88.9% (8/9)
Rated the app as good or better	85.7% (12/14)

### Clinician questionnaire

**Figure 10.**
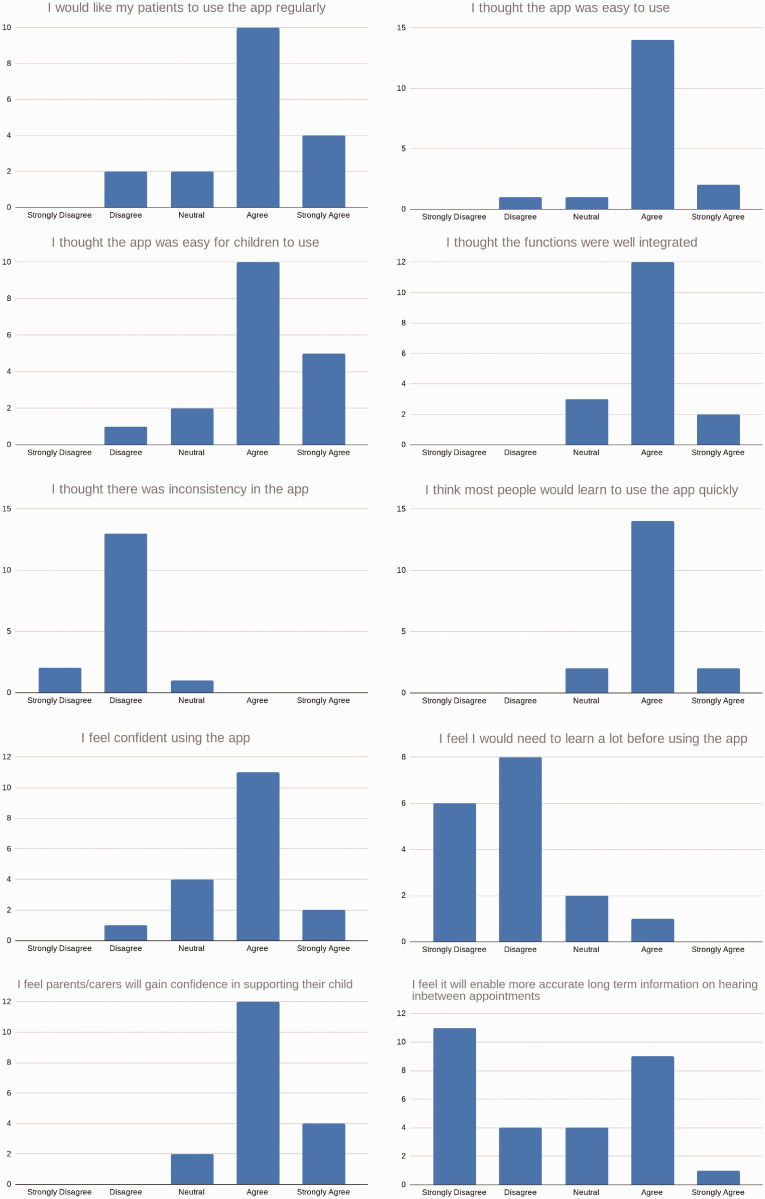
Panel of responses to the clinician questionnaire.

The clinicians’ responses to the questionnaire are summarised in Table 5 and agreement distributions for each question are summarised graphically in Figure 10.

**Table 5.** Clinician responses to the written questionnaire given immediately after being shown the app. % shows the percentage of responses where ‘agree’ or ‘strongly agree’ were selected (total number of responses – ‘neutral’, ‘disagree’ or ‘strongly disagree’.

Response	% (number who agreed/total)
Acceptability	
Felt that they would like their patients to use the app regularly	77.8% (14/18)
Felt that the information gained from the ‘How’s My Hearing?’ test would be useful to review with parents and support discussion	66.7% (10/16)
Felt that the app could enable more accurate long term information about hearing abilities between appointments	55.6% (10/18)
Accessibility	
Thought that the app was easy for adults to use	88.9% (16/18)
Thought that the app was easy for children to use	83.3% (15/18)
Thought that the app’s functions were well integrated	82.4% (14/17)
Thought that there was no inconsistency in the app	93.8% (15/16)
Thought that their patients would learn to use the app quickly	88.9% (16/18)
Felt confident using the app	72.2% (13/18)
Felt that they did not need to learn a lot before using the app	82.4% (14/17)

### Mobile Application Rating Scale (MARS)

There were a total of 10 responses, including paediatricians, audiologists, caregivers and researchers. The mean score for each category of the app’s features are shown in Table 6.

**Table 6.** Clinician/caregiver responses to the written MARS questionnaire given after being shown the app. Mean score is a mean average of all responses, which have a possible range 1–5 (1 being poorest and 5 being best).

Category	Mean score (range)
Engagement	4.8 (4.6–5)
Functionality	4.7 (4.6–5)
Aesthetics	4.7 (4.3–5)
Information	4.7 (4.3–5)
App quality	4.7 (4.4–5)

## Discussion

Despite the growing popularity of mHealth, the number of high quality applications addressing children’s speech and language development is relatively small.^17^ Potential barriers to their uptake and advancement include parental concerns about screen time and children’s compliance and ability to access them. This study addressed whether these barriers impede the uptake and acceptability of the new Hear Glue Ear app for children and their families. The initial questionnaire results demonstrate that the Hear Glue Ear app is acceptable, accessible and useful to children and families while their hearing is reduced from OME. The high number of downloads (87%) and uses between appointments (73.9%) demonstrate families’ enthusiasm for this type of support and suggest that parental concerns about screen time do not deter app uptake. These concerns may be partially assuaged by the recommendation of the app by a trusted clinician.

The high uptake of the app may also be explained by its practical design and accessibility. In this study, 93.3% of children thought the app was easy to use and 73.3% thought they could use it without an adult’s help, across the age range of 2–8 years old, for which the app was designed. This range reflects the ages with maximum prevalence of OME^7^ and confirms that the app is accessible for the most affected patient group. Children in this age group’s proficiency with using mobile apps may reflect the increasing integration of mobile and tablet-based technologies in schools. In fact, a family whose 7 year old child had a Down’s Syndrome diagnosis commented that the app was ‘too basic’, since she frequently used apps with more complex games and graphics. This contrasted with the response of an 8 year old’s family who felt that the app was well-pitched for their child. These anecdotal findings were seen repeatedly and suggest that within this age range, engagement with the app may be less dependent on chronological or development age, but rather on prior exposure to mobile applications. The majority of caregivers (82.6%) reported that their child seemed to be as engaged with the activities on the app as with other activities, which further demonstrates its appeal to children. This may be attributed to the ‘gamification’ of the app’s interface and hearing test, which is well-known to increase uptake and compliance with mHealth in children.^27^,^28^

Another perceived barrier to app use at home is families’ time availability. A 2015 study by the National Literacy Trust showed that only 29% of caregivers read to their child for more than 15 minutes per day, and this rate was influenced by factors such as parental level of education.^35^ The high rate of uptake of the Hear Glue Ear app is therefore encouraging and may be partially attributed to the app’s portability and ease of access: families reported using it most after school, before bed or during car journeys. The app provides a focussed and reliable tool to guide caregivers to support their child, which may empower families to take a more active role in their child’s speech and language development. It is known that increased parental involvement improves speech outcomes in children with hearing loss.^25^ Therefore a family-centred approach during the ‘watchful waiting’ period may significantly improve outcomes.

The majority of clinicians (77.8%) agreed that they would like their patients to use the Hear Glue Ear app regularly between appointments, demonstrating its acceptability within the OME clinical pathway. However, only 55.6% felt that the app could enable more accurate long term information about hearing abilities between appointments. In free-text spaces, clinicians explained their hesitance by concerns about conveying the outcome of the app’s hearing test as a percentage score, since parents may interpret this as percentage hearing loss, which the app has not been shown to reliably assess. To address this concern, the app display has been altered to no longer display percentage scores to parents and a safety netting pop-up message clearly states that the test is not diagnostic or intended to replace clinical audiology assessments (as shown in Figure 4). The high levels of engagement with the app suggest it could be used as a portal for speech and language therapists to upload material for families to access from home, which is a feature on the app. Technology-led practice is shown to increase children’s uptake of speech and language therapy.^18^

Comparison of the app’s game-based hearing test with clinic pure tone average (PTA) results showed a significant correlation ($r\left(22\right)=-0.656,\;p=0.000251$), which supports the validity of the app to estimate trends in hearing levels between appointments (although it is not designed to *diagnose* hearing loss). Any discrepancy which remains between the app hearing test and clinic PTA results may be explained by normal variance in PTA values, since studies have shown a +/− 10 dB variance between PTA values taken within a short time of each other is normal and expected, due to testing variation.^36^,^37^ Furthermore, the app hearing test was performed a maximum of 30 minutes following clinic assessment, which is insufficient time for fluctuations in actual hearing level to have influenced test results. The performance in the app’s test of children with the poorest hearing levels (e.g. PTA values 41.25 and 47.5 dB shown in Figure 8) may have been influenced by a ‘fatigue’ effect which has been observed to influence concentration and motivation in children with severe hearing loss.^13^,^38^ This effect could have been exacerbated by having a 30 minute audiology assessment prior to trialling the app. This effect could have been minimised by delaying the study participation to a different day or randomly allocating half of the cohort to be tested before a clinic appointment. However, all families opted for same-day participation for convenience and the authors’ rationale against trialling any families before their audiology appointment was to prevent affecting children’s performance in their diagnostic audiology test and thus to avoid any impact of the research on clinical care.

This study demonstrates that, despite the perceived barriers to the uptake of mHealth in the paediatric population, the Hear Glue Ear app is acceptable and accessible to children, their caregivers and clinicians working in the field, and its quality is verified by the MARS assessment. The app’s game-based hearing test is also validated as a means of estimating trends in a child’s hearing level between appointments. The accessibility of the app depends on access to Wifi and ability to navigate its user interface, and is unaffected by geographical location or ability to pay. In scenarios where face-to-face appointments are limited, as in the current COVID-19 pandemic, the app may provide ongoing support and continuity of care to families remotely. There is also the potential for use in low resource settings, where it may provide an estimate of changing hearing levels and support speech and language development in communities with limited access to audiology services. Furthermore, there is the future possibility of using the app in conjunction with novel technologies such as bone-conducting headsets via Bluetooth, to further improve access and benefit for children with the most severe conductive hearing losses.^39^ A considerable advantage to the use of mobile health apps is the ability to update and improve in line with best practice, at minimal additional cost or inconvenience to end users. Following increasing appreciation of these benefits, technology in the field of paediatric audiology is rapidly evolving and exciting new innovations have the potential to support and guide family-centred care within management pathways, as NICE advocates.^12^
